# Cigarette Smoking and *p16^INK4α^* Gene Promoter Hypermethylation in Non-Small Cell Lung Carcinoma Patients: A Meta-Analysis

**DOI:** 10.1371/journal.pone.0028882

**Published:** 2011-12-13

**Authors:** Bo Zhang, Wei Zhu, Ping Yang, Tao Liu, Mei Jiang, Zhi-Ni He, Shi-Xin Zhang, Wei-Qing Chen, Wen Chen

**Affiliations:** 1 Department of Toxicology, Guangdong Provincial Key Laboratory of Food, Nutrition and Health, School of Public Health, Sun Yat-sen University, Guangzhou, China; 2 Department of Toxicology, Guangzhou Center for Disease Control and Prevention, Guangzhou, China; 3 Faculty of Biostatistics and Epidemiology, School of Public Health, Sun Yat-sen University, Guangzhou, China; 4 Guangdong Institute of Public Health, Guangzhou, China; 5 Center for Disease Control and Prevention of Guangdong Province, Guangzhou, China; University Clinic of Navarra, Spain

## Abstract

**Background:**

Aberrant methylation of promoter DNA and transcriptional repression of specific tumor suppressor genes play an important role in carcinogenesis. Recently, many studies have investigated the association between cigarette smoking and *p16^INK4α^* gene hypermethylation in lung cancer, but could not reach a unanimous conclusion.

**Methods and Findings:**

Nineteen cross-sectional studies on the association between cigarette smoking and *p16^INK4α^* methylation in surgically resected tumor tissues from non-small cell lung carcinoma (NSCLC) patients were identified in PubMed database until June 2011. For each study, a 2×2 cross-table was extracted. In total, 2,037 smoker and 765 nonsmoker patients were pooled with a fixed-effects model weighting for the inverse of the variance. Overall, the frequency of *p16^INK4α^* hypermethylation was higher in NSCLC patients with smoking habits than that in non-smoking patients (OR = 2.25, 95% CI = 1.81–2.80). The positive association between cigarette smoking and *p16^INK4α^* hypermethylation was similar in adenocarcinoma and squamous-cell carcinoma. In the stratified analyses, the association was stronger in Asian patients and in the studies with larger sample sizes.

**Conclusion:**

Cigarette smoking is positively correlated to *p16^INK4α^* gene hypermethylation in NSCLC patients.

## Introduction

The incidence of lung cancer is increasing worldwide, particularly in developing countries. In China the death rate of lung cancer has been increasing from 7.1 to 30.8 per 100,000 during 1975–2005. People dying due to lung cancer accounted for 23% of total amount of cancer death in 2005 [1]. 80% of primary lung cancers are non-small cell lung carcinoma (NSCLC) which is characterized by a long asymptomatic latency and poor prognosis. Without an early diagnostic approach, over 40% of lung cancer patients develop metastasis at the time of diagnosis and survive for a short time period under a conventional chemotherapy [2]. Only 15% of NSCLC patients can survive over 5 years [3]. Thus, it is essential to identify biomarkers for early prediction of lung cancer.

Cigarette smoking is a well known driving force for lung cancer development. The lifetime risk of developing lung cancer is 17.2% in male smokers and 11.6% in female smokers, which is much higher than that in nonsmokers with 1.3% in male and 1.4% in female [4]. Although most lung cancers are associated with cigarette smoking, it is statistically estimated that 15% of them in males and 53% in females, accounting for about 25% of all lung cancers, are not attributable to cigarette smoking [3]. Lung cancers arising in nonsmokers are more frequently adenocarcinomas, affect females disproportionately more than males, and have regional differences ranging from 10–15% in Europe and North America to 30–40% in Asian countries [5]–[7]. Moreover, nonsmoker lung cancers have improved survival and are more sensitive to epidermal growth factor receptor (EGFR) tyrosine kinase inhibitor therapy. It might be due to that the activation of EGFR by gene mutations appears more often in nonsmoker lung cancers [5], [6]. Taken together, lung cancer in nonsmokers would probably be considered a separate cancer category. If so, it would rank as the seventh most common cause of cancer death worldwide [3]. However, whether these clinical-pathological and molecular differences between lung cancer in nonsmokers and smokers are related to cigarette smoking is still unknown.

The interest in cancer-associated changes in gene methylation has grown enormously in recent years with the speculation that the promoter methylation status may provide an early biomarker for tumorigenesis [8]. Silencing of genes by aberrant promoter hypermethylation has been recognized as a key event in cancer initiation and progression [9], [10]. Highly sensitive assays such as methylation-specific PCR (MSP), which could detect one methylated allele in the presence of 10^3^–10^4^ unmethylated alleles [11], [12], have been used to assess gene-promoter methylation in primary tumors, serum, plasma, sputum or specimens from the aerodigestive tract epithelium [13]. Numerous studies have investigated the methylation statuses of specific genes in body fluids and tumor tissues of lung cancer patients, and identified more than 60 genes as being epigenetically silenced in lung tumors [8]. The proof-of-concept studies suggested that gene-specific promoter methylation occurs as an early event in lung cancer. For example, hypermethylation of *p16^INK4α^* (also known as cyclin-dependent kinase inhibitor 2A, *CDKN2A*) or O^6^-methylguanine-DNA methyltransferase (*MGMT*) was found in sputum samples 5 to 36 months prior to clinical diagnosis [14]. Moreover, the frequency of *p16^INK4α^* hypermethylation increased progressively from 17% in basal-cell hyperplasia to 24% in squamous metaplasia, and to 60% in squamous cell carcinomas [15]. The correlation between gene methylation and recurrence of lung cancer has also been reported [16], [17]. The promoter hypermethylation of several genes including *p16^INK4α^*, cadherin 13 (*CDH13*), Ras association (RalGDS/AF-6) domain family member 1 (*RASSF1A*) and adenomatous polyposis coli (*APC*) in NSCLC specimens is associated with early recurrence after surgery [17]. Moreover, the reversibility of DNA methylation modification makes it possible that the expression of genes that have undergone epigenetic silencing becomes reactivated by the inhibitors of DNA methylation. Many agents such as 5-azacytidine and zebularine are currently being tested in clinical trials [18]. Despite the significance of gene promoter methylation in predicting incidence or prognosis and in epigenetic therapy of lung cancer, the manner in which these epigenetic lesions accumulate during carcinogenesis is not completely understood. The missing links among environmental factors, DNA methylation changes, and lung cancer limit the applications of methylated genes as a biomarker for early detection of lung cancers.

The correlation between cigarette smoking and aberrant gene methylation has been extensively studied, but the results are inconsistent and inconclusive. The present study mainly focused on *p16^INK4α^* gene as it is the first gene identified in lung cancer, and is transcriptionally silenced predominantly through aberrant promoter hypermethylation [19]. Here, we performed a literature-based systematic review and meta-analysis to quantitatively analyze the correlation between cigarette smoking and *p16^INK4α^* gene methylation in NSCLC patients.

## Methods

### Ethics Statement

An ethics statement was not required in this study.

### Literature search

We systematically searched for all published articles indexed in PubMed database from 1966 to June 18, 2011 with the Medical Subject Headings (MeSH) and corresponding free text: “smok* AND (p16* OR CDKN2A OR INK4A) AND (methylation OR epigene*)”. We also manually searched the references of these publications in order to retrieve additional studies. Only those published as full-text articles and in English or in Chinese were included as candidates.

### Inclusion and exclusion criteria

Studies were selected for analysis if they met the following criteria: 1) they were original epidemiological studies on the correlation between cigarette smoking and *p16^INK4α^* methylation; 2) they were conducted in lung cancer patients; 3) the specimens used for methylation analysis must include surgically resected primary tumor samples, while other specimens such as sputum, serum, bronchial lavage samples and normal or non-malignant lung tissue may be used, but not essential; 4) *p16^INK4α^* methylation status was examined using methylation-specific PCR (MSP) or quantitative MSP (QMSP); 5) the subjects in every study comprised nonsmokers and smokers (former smokers and/or current smokers), irrespective of minor discrepancies of the definition of nonsmokers over all studies. To avoid duplication of data, we carefully checked the author names, research institutions and procedures for enrolling participants. Where several publications reported from the same population data, only the most rounded study with more information was included.

### Data collection

For each eligible study, we collected information regarding authors, year and source of publication, country of origin, inclusion criteria, exclusion criteria, histology of lung cancer, types of biological specimen, number of participants, participants' age and gender, smoking behaviour, *p16^INK4α^* methylation frequencies in nonsmokers and smokers and the method for methylation detection. All included studies used nonsmokers as a control group, though some of them did not provide the definition of nonsmoker. In studies defining nonsmoker, there were three different definitions of nonsmoker: (1) daily cigarette consumption×years of smoking = 0; (2) less than 100 cigarettes in entire lifetime; (3) less than 20 pack-years. Since it is impossible to redefine nonsmoker based on a unified standard, we combined nonsmokers in our meta-analysis according to their original group in each individual study. Correspondingly, subjects except nonsmokers were smokers comprising former smokers and current smokers, and light, moderate or heavy smokers. Then data was integrated in 2×2 tables demonstrating the methylation/unmethylation of *p16^INK4α^* gene according to the cigarette smoking status (smoker/nonsmoker). All data were extracted independently by two reviewers using a standard form. Minor discrepancies were resolved by the authors' discussion.

### Meta-analysis and statistical analysis

The foremost analysis examined the differences in the frequency of *p16^INK4α^* methylation in lung cancer tissues between smoker and nonsmoker patients. Summary OR was obtained across all studies. Heterogeneity was examined using the I^2^ statistic, which represents the proportion of variation in the effect sizes that is attributable to true differences across studies rather than to a random error. Results without heterogeneity were pooled using the fixed-effect model, following the Mantel-Haenszel method. Otherwise, the random effect analysis with the method of DerSimonian and Larid was used.

The meta-analyses were performed using Stata statistical software (version 10.0, Stata Corporation, College Station, Texas, USA). Results were shown in forest plots, where the sizes of the boxes for individual studies were inversely proportional to the variances of the log relative risks, and the horizontal lines represent 95% confidence interval (CI).

The frequencies of *p16^INK4α^* methylation in smokers and nonsmokers were compared by Wilcoxon signed rank test. The coefficients of Spearman's rank correlation were calculated between frequency of *p16^INK4α^* methylation and sample size. These analyses were performed using SPSS for Windows (version 11.5, SPSS Inc., Chicago, IL, USA).

## Results

### Study characteristics

**Figure 1:**
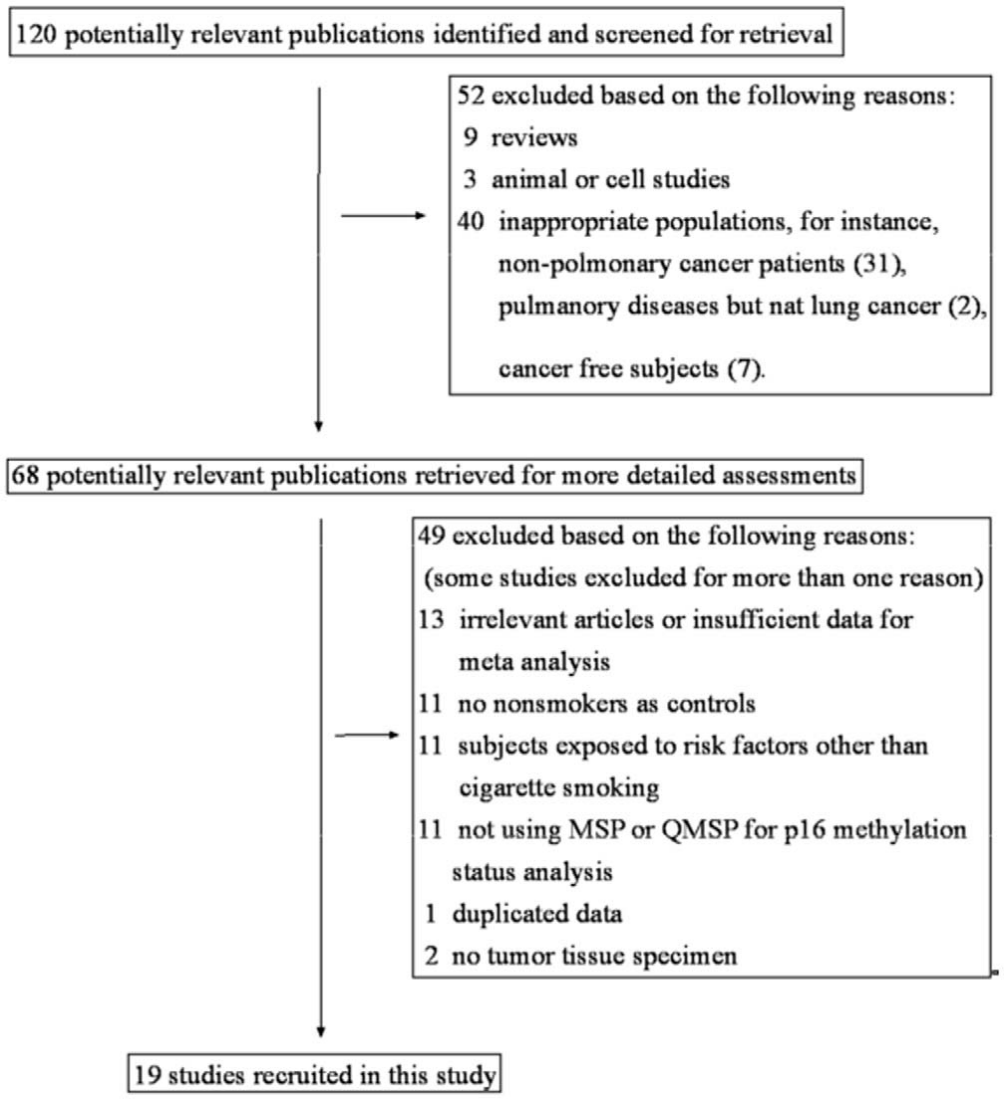
Flow diagram of the stepwise selection from associated studies.

Following the inclusion and exclusion criteria described above, 19 studies [12], [20]–[37] were included in the analysis (Figure 1). The characteristics of these studies are summarized in Table 1. Of these 19 studies, eight defined the subtypes (adenocarcinoma or squamous cell carcinoma) of NSCLCs. Ten studies were conducted in Asia (2 in China, 6 in Japan and 2 in Korea), five were in USA, and the remaining four were in Australia, Greece, Chile, and multi-areas in Asia-Pacific regions (USA, Australia, Japan and Taiwan) respectively (Table 1). In two studies [12], [25], lung adenocarcinoma (AC) patients and squamous cell carcinoma (SCC) patients were analyzed separately, therefore they were treated as separate items in the meta-analysis. As for the primer sequences of MSP, 15 studies used the same primers designed by Herman et al. in 1996 [11]. The primer sequences for detecting methylated *p16^INK4α^* gene were 5′-TTA TTA GAG GGT GGG GCG GAT CGC-3′ (sense) and 5′-GAC CCC GAA CCG CGA CCG TAA-3′ (antisense). The size of the PCR product for the methylated reaction was 150 bp. The primer sequences used for the unmethylated promoter were 5′-TTA TTA GAG GGT GGG GTG GAT TGT-3′ (sense) and 5′-CAA CCC CAA ACC ACA ACC ATA A-3′ (antisense). The size of the PCR product for the unmethylated reaction was 151 bp.

**Table 1:** Characteristics of studies on the overall relationship between cigarette smoking and p16 methylation in lung cancer patients.

First author	Year	Location	Histology	Age(y)	Sample size(n)	p16 methylation in smoker	p16 methylation in nonsmoker
Sanchez-Cespedes [19]	2001	USA	NSCLC	66±3	47	7/33	5/14
Zochbauer-Muller [20]	2001	Australia	NSCLC	28–81	107	27/98	0/9
Kim [21]	2001	USA	NSCLC	67±11	185	49/172	2/13
Yanagawa [22]	2002	Japan	NSCLC	67±2	51	13/37	1/14
Toyooka [12]	2003	Asia-Pacific	AC	26–87	295	47/183	6/112
Toyooka [12]	2003	Asia-Pacific	SCC	26–87	189	58/172	5/17
Yanagawa [23]	2003	Japan	NSCLC	39–86	75	21/55	2/20
Toyooka [24]	2004	Japan	AC	No data	217	29/120	10/97
Toyooka [24]	2004	Japan	SCC	No data	138	46/130	1/8
Kim [25]	2004	Korea	SCC	No data	125	37/117	3/8
Divine [26]	2005	USA	AC	33–86	203	81/157	18/46
Liu [27]	2006	USA	NSCLC	65±10	122	51/81	13/41
Nakata [28]	2006	Japan	NSCLC	40–85	202	38/139	9/63
Toyooka [29]	2006	Japan	AC	No data	164	24/86	7/78
Georgiou [30]	2007	Greece	NSCLC	45–75	27	20/24	2/3
Guzman [31]	2007	Chile	NSCLC	66±9	65	39/54	10/11
Kim [32]	2007	Korea	NSCLC	41–82	99	18/79	4/20
Tessema [33]	2009	USA	AC	66	175	67/100	48/75
Wang [34]	2010	China	AC	46–84	56	9/20	10/36
Yanagawa [35]	2011	Japan	AC	39–86	62	7/36	2/26
Zhang [36]	2011	China	NSCLC	32–79	198	82/144	18/54

### Combined results and subgroup analyses

**Figure 2:**
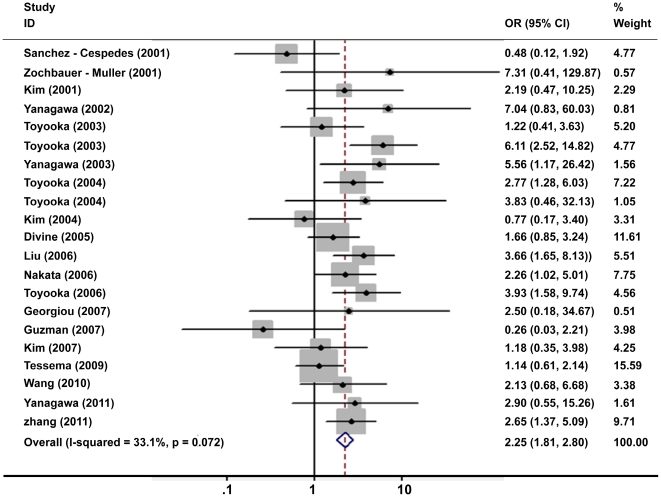
Meta-analysis of cigarette smoking and *p16^INK4α^* methylation in all NSCLC patients.

**Figure 3:**
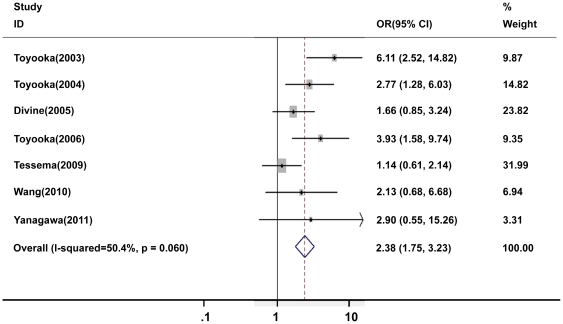
Meta-analysis of cigarette smoking and *p16^INK4α^* methylation in lung adenocarcinoma patients.

In general, the frequencies of *p16^INK4α^* methylation ranged from 19 to 83% (median 34%) in smoker patients, which were much higher than those in nonsmoker patients with range from 0 to 91% (median 20%). In the meta-analysis, 2802 NSCLC patients including 2037 smokers and 765 nonsmokers were included in pooling the overall correlation estimation. Under the fixed-effects model, the pooled odds ratio (OR) of *p16^INK4α^* methylation in smoker patients, compared to nonsmoker patients, was 2.25 with 95% CI = 1.81–2.80 (Figure 2). Of six studies only including AC patients, the subgroup analysis showed no significant difference in the association between cigarette smoking and *p16^INK4α^* methylation when comparing AC patients (OR = 2.38, 95%CI = 1.75–3.23) (Figure 3) and all NSCLC patients (OR = 2.25, 95% CI = 1.81–2.80).

**Figure 4:**
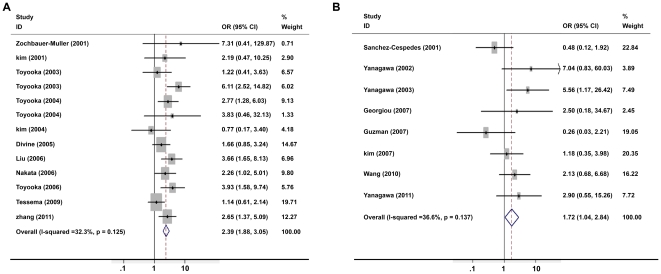
Meta-analysis of cigarette smoking and *p16^INK4α^* methylation in NSCLC patients stratified by sample size. A: sample size >100. B: sample size <100. Stratification by sample size showed a stronger association in studies with a relatively larger sample size.

**Figure 5:**
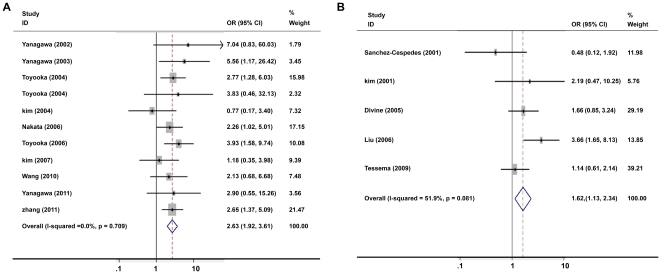
Meta-analysis of cigarette smoking and *p16^INK4α^* methylation in NSCLC patient stratified by study region. A: Asian studies. B: North American studies. The association between cigarette smoking and *p16^INK4α^* methylation tended to be stronger in Asian studies compared to the North American studies.

Because there was a borderline significant but moderate degree of heterogeneity among the 19 studies (I^2^ = 33.1%, *P* = 0.072), we performed sensitivity analyses to identify potential sources of heterogeneity. Stratification by sample size showed a stronger association in larger-size (>100 patients per study) studies (OR = 2.39, 95% CI = 1.88–3.05, Figure 4A) than that in smaller-size studies (OR = 1.72, 95% CI = 1.04–2.84, Figure 4B). Stratified analysis also revealed that the associations varied among the subjects from different regions. The association between cigarette smoking and *p16^INK4α^* methylation tended to be stronger in 9 Asian studies (OR = 2.63, 95%CI = 1.92–3.61) (Figure 5A) compared to the 5 North American studies (OR = 1.62, 95%CI = 1.13–2.34) (Figure 5B). In addition, there was no inter-study heterogeneity in Asian studies (I^2^ = 0, *P* = 0.709).

Four of the 19 included studies [12], [21], [23], [31] and another two excluded studies [38], [39] had compared the frequencies of *p16^INK4α^* methylation in adjacent noncancerous tissues or sputum specimens from NSCLC patients with or without smoking habits and no significant differences were found (Table S1). In another 7 excluded studies [40]–[46], *p16^INK4α^* methylation was examined in sputum, bronchial lavage samples or blood specimens from cancer-free subjects. We failed to find a link between cigarette smoking and the frequency of *p16^INK4α^* methylation (Table S2, Figure S1). These results suggested that cigarette smoking had no impact on *p16^INK4α^* hypermethylation in the surrogate samples from NSCLC patients, and that the positive association between cigarette smoking and *p16^INK4α^* hypermethylation was not present in health conditions.

### Publication bias

**Figure 6:**
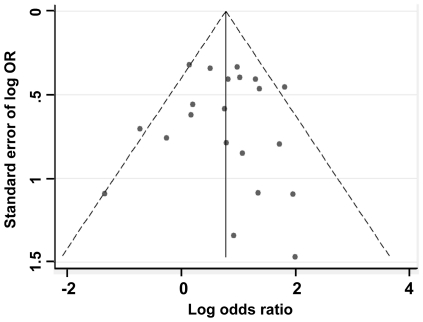
Begg's funnel plot for visual assessment of the presence of publication bias for all studies included in the meta-analysis (each study is represented by an open circle).

To ensure the quality of this study, we performed a Begger's funnel plot and Egger's tests to eliminate the publication bias of included studies. As shown in Figure 6, the shapes of the funnel plots showed a little asymmetry at the bottom. However, Egger's test, which provided statistical data of funnel plot symmetry, did not show any evidence of publication bias (t = 0, *P* = 0.998).

## Discussion

The present study, based on the accumulated evidences from 19 cross-sectional studies, indicates that cigarette smoking is positively related to *p16^INK4α^* hypermethylation in tumor tissues from NSCLC patients. The frequency of *p16^INK4α^* methylation in smoker lung cancer patients was 2.25 times higher than that in nonsmoker patients. The association appeared to be stronger in Asian patients and in studies with a larger number of subjects, but without a histology (AC or SCC) specificity. However, this positive correlation did not exist in adjacent noncancerous tissues from NSCLC patients and in biological specimens of ‘healthy’ subjects without cancer. Given the results that cigarette smoking leading to *p16^INK4α^* hypermethylation was related to the stage progression of tumorigenesis, we speculated that *p16^INK4α^* hypermethylation might be an early marker for cancer diagnosis, particularly in cigarette smoking patients.

Recent epidemiological studies have revealed that molecular mechanisms underlying the development of lung cancers differed between nonsmokers and smokers. For instance, *EGFR* pathway is frequently activated by gene mutations in nonsmoker lung cancers, while mutations of *KRAS* often occur in smoker lung cancer. However, the mutations in either gene in lung adenocarcinomas are rarely seen although the biological consequences of KRAS and EGFR mutations share similarities in regulation of cell proliferation, survival and apoptosis [47], [48]. In the present study, we demonstrate that the frequency of *p16^INK4α^* hypermethylation is slightly but significantly higher in smoker patients than that in nonsmoker patients. It is well known that *p16^INK4α^* plays an essential role in the development of most human cancers for the reason that the p16/cyclinD1/CDK4/RB signaling pathway controls the cell cycle at the G1/S transition [49]. Hypophosphorylated RB inhibits G1/S transition by binding to E2F1 transcription factor and exerts its tumor-suppressor function. Once hyperphosphorylated by the cyclinD1/CDK4 complex, RB releases E2F1, which results in transition from G1 to S phase. p16 prevents RB from phosphorylation by inhibition of CDK4, leading to a cell cycle arrest. Suppression of p16 expression allows unregulated phosphorylation of the RB protein and leads to uncontrolled cell cycle progression and cell division [50]. *p16^INK4α^* has a low frequency of mutations in lung cancer [51]. Its inactivation is mainly through gene promoter hypermethylation [19]. For example, Nakata et al. found that in tumors with *p16^INK4α^* hypermethylation, 63.3% showed reduced expression; whereas, in tumors without *p16^INK4α^* hypermethylation, only 33.7% showed reduced expression (*P* = 0.0002) [29]. The positive correlation between cigarette smoking and *p16^INK4α^* hypermethylation demonstrates that cigarette smoking plays an important role in determining the molecular signatures involved in lung cancer development.

The mechanism for cigarette smoking inducing gene-specific hypermethylation, e.g. *p16^INK4α^*, remains unclear. *De novo* methylation uses S-adenosyl-methionine as a methyl donor and adds a methyl group to the cytosine ring to form methyl cytosine, which is catalyzed by DNA methyltransferases (DNMT) 1, 3a, or 3b [52]. It is estimated that DNMT1 is responsible for about 90% of methyltransferase activity in mammalian cells [53]. DNMT1 overexpression was found in many types of cancers including lung cancers, particularly in patients who were smokers [54]–[56]. A recent study found that DNMT1 was highly expressed in tumor tissues in a dose response manner compared with the non neoplastic stroma tissues, not only in tobacco-specific carcinogen nicotine-derived nitrosamine ketone (NNK)-induced mouse lung cancer but also in human lung cancer associated with cigarette smoking [57]. Moreover, it was demonstrated that NNK increased DNMT1 expression and activity by blocking its degradation related to ubiquitin-proteasome [57]. AKT/GSK3β/βTrCP signaling is implicated in the accumulation of nuclear DNMT1, which leads to hypermethylation of *p16^INK4α^*, fragile histidine triad gene (*FHIT*) and retinoic acid receptor β (*RARB*), and ultimately leads to tumorigenesis and poor prognosis [57]. Although the direct interaction of DNMT1 to the *p16^INK4α^* gene promoter is not yet characterized [58], these findings indicated that tobacco-induced DNMT1 overexpression might be responsible for maintaining the hypermethylation status of *p16^INK4α^* gene.

Lung cancer in nonsmokers is now a prominent public health concern. However, the major causes of them have yet not been identified. Environmental tobacco smoke (ETS), for example, second-hand smoke, has been recognized as a high risk factor [59]–[62]. According to the report from International Agency for Research on Cancer (IARC), the risk for developing lung cancer from ETS exposure might reach 35% in men and 25% in women [63]. Given that cigarette smoking has a cause-effect on *p16^INK4α^* hypermethylation, ETS exposure may explain, at least partly, the variable percentage of *p16^INK4α^* hypermethylation in nonsmoker patients. Other factors such as exposed to asbestos, chromium, arsenic, cadmium, silica, or nickel, or outdoor air pollutants, previous lung disease, and dietary factors have also been implicated in non smoking-related risk [5], [6]. But so far there is still a missing link between environmental factors, *p16^INK4α^* hypermethylation, and lung cancer, which limits the use of gene-specific hypermethylation as a biomarker to detect lung cancer in early stage. Thus, the molecular mechanism underlying lung cancer, irrespective of tobacco-association, should be further elucidated.

In this study, we observed that the frequency of *p16^INK4α^* hypermethylation in NSCLC patients varied among different studies. The combined frequency in the present meta-analysis was less than 35%. The discrepancy and the relative low frequency might be due to the method used for detection of methylation, the variation in defining cigarette smokers, and insufficient information of clinical outcome. Although MSP is sufficiently sensitive, the conditions of PCR may affect the results to a large extent. The results seemed to be a little artificial particularly when PCR reaction was performed using both methylated and unmethylated primers. As for definition of cigarette smoker or nonsmoker, it lacked a consistent criterion followed by each investigation. In addition, current smokers and former smokers were not clearly distinguished, and the quantity of smoking was not calculated in the meta-analysis due to limited data. Moreover, insufficient clinical information such as the stage of NSCLC made it difficult to predict the prognosis based on the results provided.

In conclusion, cigarette smoking is suggested to be positively related to *p16^INK4α^* methylation in human NSCLC, highlighting the potential importance of *p16^INK4α^* promoter methylation in early cancer diagnosis. Furthermore, it is well known that the risk for developing lung cancer in smokers is 8 to 13 times higher than that in nonsmokers, while the risk of *p16^INK4α^* hypermethylation in lung cancer patients with smoking habits was only 2.2 times increased than that in nonsmoker patients, we speculate that many other aberrant epigenetic modifications, together with the genetic damage are involved in lung cancer development, which needs to be addressed in further investigation.

## Supporting Information

Figure S1 Meta-analysis of cigarette smoking and *p16^INK4α^* methylation in noncancerous patients.
(TIF)

Table S1 Characteristics of studies on the correlations between cigarette smoking and *p16^INK4α^* methylation in noncancerous tissue from cancer patients.
(DOC)

Table S2 Characteristics of studies on the correlation between cigarette smoking and *p16^INK4α^* methylation in noncancerous patients.
(DOC)
