# Supplementary figures and images for: Cigarette Smoking and p16INK4α Gene Promoter Hypermethylation in Non-Small Cell Lung Carcinoma Patients: A Meta-Analysis
Source: PLoS One. 2011 Dec 13;6(12):e28882. doi: 10.1371/journal.pone.0028882 (PMC3236763; doi:10.1371/journal.pone.0028882)

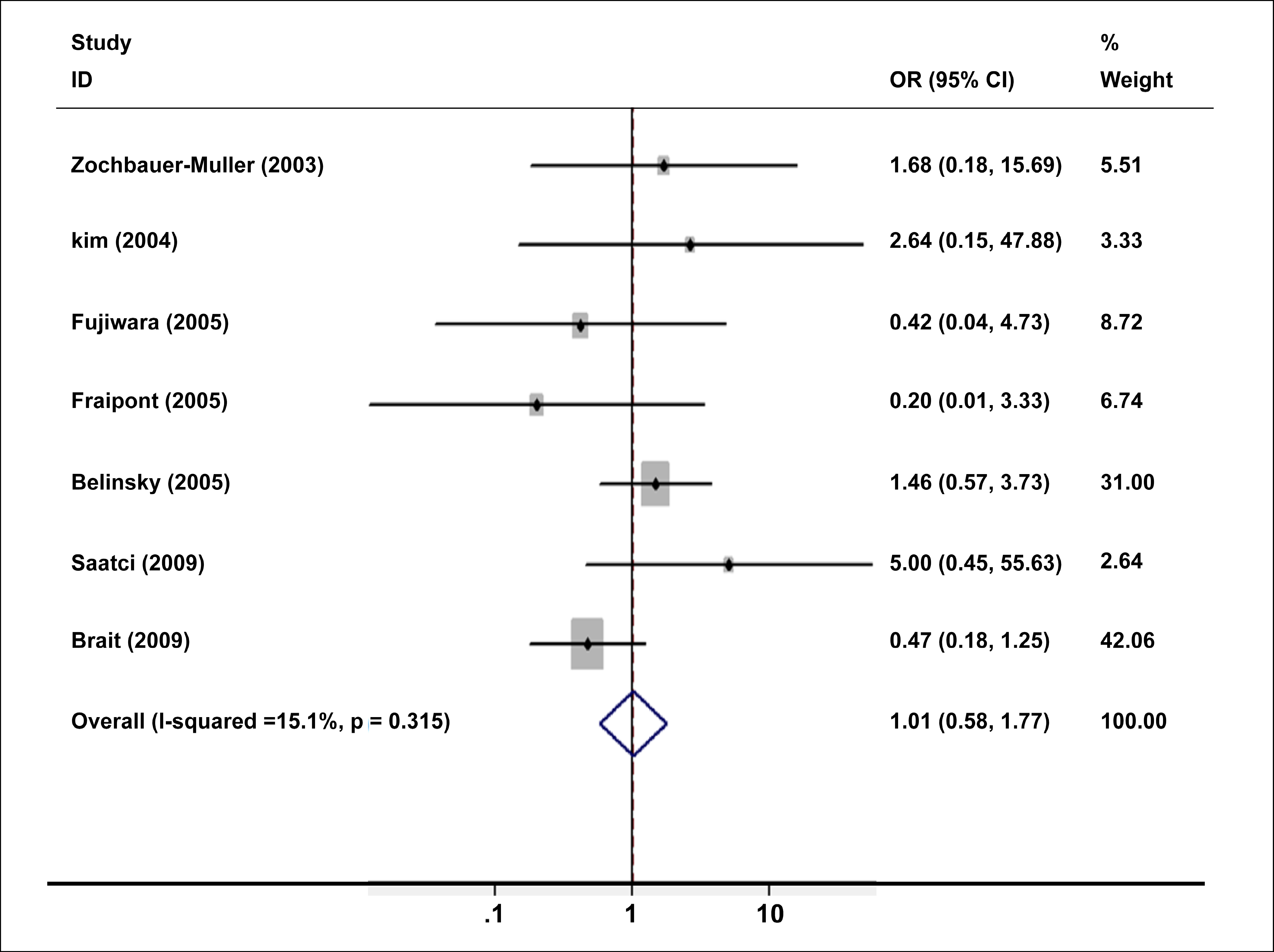

Supplement: Figure S1 — Meta-analysis of cigarette smoking and p16INK4α methylation in noncancerous patients. (TIF) [file pone.0028882.s001.tif]
