# Supplementary material for: Cigarette Smoking and p16INK4α Gene Promoter Hypermethylation in Non-Small Cell Lung Carcinoma Patients: A Meta-Analysis
Source: PLoS One. 2011 Dec 13;6(12):e28882. doi: 10.1371/journal.pone.0028882 (PMC3236763; doi:10.1371/journal.pone.0028882)
Supplement: Table S1 — Characteristics of studies on the correlations between cigarette smoking and p16INK4α methylation in noncancerous tissue from cancer patients. (DOC) [file pone.0028882.s002.doc]

| Table S1 Characteristics of studies on the relationships between cigarette smoking and p16 methylation in noncancerous tissue from cancer patients | | | | | | | | |
| --- | --- | --- | --- | --- | --- | --- | --- | --- |
| First author | Year | Location | Histology | Specimen | Age  (y) | Sample size  (n) | p16 methylation  in smoker  (n) | p16 methylation  in nonsmoker  (n) |
| Zochbauer-Muller | 2001 | Australia | NSCLC | adjacent noncancerous tissue | 28-81 | 104 | 0/95 | 0/9 |
| Yanagawa | 2002 | Japan | NSCLC | adjacent noncancerous tissue | 67±2 | 51 | 0/37 | 0/14 |
| Toyooka | 2003 | Asia-pacific | NSCLC | adjacent noncancerous tissue | 26-87 | 84 | 4/67 | 0/17 |
| Georgiou | 2007 | Greece | NSCLC | adjacent noncancerous tissue | 45-75 | 27 | 12/24 | 1/3 |
| konno | 2004 | Japan | NSCLC | sputum | 42-86 | 70 | 12/55 | 3/15 |
| Peng | 2010 | China | NSCLC | sputum | 56.6 ± 9.4 | 82 | 28/52 | 12/30 |
