# Supplementary material for: Cigarette Smoking and p16INK4α Gene Promoter Hypermethylation in Non-Small Cell Lung Carcinoma Patients: A Meta-Analysis
Source: PLoS One. 2011 Dec 13;6(12):e28882. doi: 10.1371/journal.pone.0028882 (PMC3236763; doi:10.1371/journal.pone.0028882)
Supplement: Table S2 — Characteristics of studies on the correlation between cigarette smoking and p16INK4α methylation in noncancerous patients. (DOC) [file pone.0028882.s003.doc]

| Table S2 Characteristics of studies on the relationships between cigarette smoking and p16 methylation in noncancerous patients | | | | | | | | | |
| --- | --- | --- | --- | --- | --- | --- | --- | --- | --- |
| First author | Year | Country | Subjects | Age | sex | Specimen | Sample size | p16 methylation  in smoker (n) | p16 methylation  in nonsmoker (n) |
| Zochbauer-Muller | 2003 | Canda | cancer free subjects | 21-75 | F+M | sputum | 103 | 4/73 | 1/30 |
| kim | 2004 | Korea | cancer free subjects | 26-82 | F+M | bronchial lavage | 127 | 8/110 | 0/17 |
| Fujiwara | 2005 | Japan | nonmalignant pulmonary diseases | 26-89 | F+M | serum | 100 | 1/54 | 2/46 |
| Fraipont | 2005 | France | patients with a high risk for NSCLC | No data | No data | bronchial lavage | 122 | 20/120 | 1/2 |
| Belinsky | 2005 | USA | cancer-free women | 40-76 | F | plasma | 195 | 16/121 | 7/74 |
| Saatci | 2009 | Turkey | healthy subjecys | mean | No data | blood | 28 | 3/12 | 1/16 |
| Brait | 2009 | USA | healthy subjecys | 40-90 | M | blood | 157 | 16/121 | 7/36 |
